# Discussion on Uterine Hemorrhages

**Published:** 1894-01

**Authors:** 


### DISCUSSION ON DR. KEILLER'S PAPER ON "UTERINE HEMORRHAGES"

Dr. H. A. West.—You have, Mr. President, very thoroughly presented the causes and treatment of uterine hemorrhages. I have very little to add, except to emphasize the importance and frequency of abortion especially when artificially produced, as a cause of hemorrhage and subsequently endometritis. Abortion, pursuing a normal course, that is to say, when there is a complete separation and perfect expulsion of the placenta, is comparatively easy to manage. Quietude in bed for a sufficient length of time to insure involution of the uterus, contraction of which can be stimulated by moderate doses of ergot, with attention to the bowels, are the simple measures required; but there are a certain number of cases, and they are apt to recur repeatedly in the same woman, when the fœtus is expelled, yet the after-births remain firmly, but partially attached, and there is furious bleeding from the open mouths of some uterine sinuses. What are we to do under such circumstances? The answer to this question is not always easy. The paramount indication, you will say, is to remove the retained placenta which is the cause of the hemorrhage; but how and when is this to be done? Who has not experienced the difficulty in extracting a placenta or an ovum when the os is contracted, so as barely to admit one finger? The difficulty is still greater when there is more or less complete detachment: If one gives chloroform and attempts to break up and remove piece-meal with finger or curette, the foreign matter, portions are likely to be left behind, and are a certain source of future trouble. I believe it is better practice, unless we are certain we can remove the entire uterine contents, to tampon the womb as well as the vagina thoroughly with iodoform gauze, which will control the bleeding, and give a little time for nature to separate the adherant mass from the uterine wall, when it will often be spontaneously expelled, and if not, its removal with finger, forceps or curette becomes a comparatively easy matter.

Dr. A. F. Sampson:—The discussion of Dr. Keiller's exhaustive paper under the broad title of "Uterine Hemorrhages," would be too much for us to undertake at one sitting. I am especially pleased to see the remarks that have been made dwell upon ectopic pregnancy, as this is a condition that has recently concerned me very much. I cannot agree with those who claim ectopic pregnancy as so easily and rapidly diagnosed. As my experience goes to show, I will admit that when this form of pregnancy has been going on for a couple of months, or rupture of the sack, with its attending shock takes place, it is an easy matter to diagnose; but not so in the first few weeks of ectopic gestation. To illustrate: I was called to a case at midnight, the latter part of September. There was no evidence of any shock. The patient gave a history of delayed abortion. She had gone but a few days over her menstruation, had uterine pains and some hemorrhage for the past three or four days. From what I could gather, I believed the product had passed, and the pains that were referred to the uterus were due to the partially retained placenta, etc. I prescribed ergot, quinine and hot vaginal douches. There was no abnormal temperature; the pulse was in the eighties, and no evidence of a serious condition was manifested. The patient seemed to progress satisfactorily for several days, though slight hemorrhages kept up. About twenty days after my first visit, the patient not doing satisfactorily as to the flow, which still kept up and began to have an offensive odor, I had chloroform administered, dilated the cervix and curetted. Before doing this I made a careful examination and detected no evidence of pelvic trouble, except what I attributed to the endometrium. My patient seemed to have no immediate bad effects from the curetting; in fact, seemed to improve to such a degree that I, in due time, allowed her to sit up. Later, pelvic cellulitis began to set up, that proved obstinate to treatment. A consultation was had, and my diagnosis of a pelvic cellulitis was confirmed, as by now a mass had developed in the right pelvic region. Later still, this case passed out of my hands and she became the patient of a most skilled surgeon, whom I had kept well informed as to the progress of the case. Shortly after taking charge of this case I learned that the attending physician suspecting that pus had developed an abscess which seemed to point towards the vault of the vagina, he opened up what he expected to find a pulvic abscess, when, lo! a gush of blood with a profuse hemorrhage following proved to be a case of ectopic pregnancy. This case taught me a lesson.

Dr. J. E. Thompson.—I have seen a case of extra-uterine pregnancy at the 4th month, in which all the signs of pregnancy but that of hemorrhage were present. A complete decidua was passed at the second month. A tumor was found occupying a position to the left of the uterus; it was freely movable, of the size of an orange, and could be completely explored An operation was done at the fourth month, and the tumor completely removed without difficulty. It proved to be a pregnancy in an ovarian sac, no foetus being present, but placental villi were found microscopically.

